# Cytological diagnosis of adult-type fibrosarcoma of the neck in an elderly patient. Report of one case and review of the literature

**DOI:** 10.1186/1471-2482-13-S2-S42

**Published:** 2013-10-08

**Authors:** Immacolata Cozzolino, Alessia Caleo, Vincenzo Di Crescenzo, Mariapia Cinelli, Chiara Carlomagno, Alfredo Garzi, Mario Vitale

**Affiliations:** 1 Department of Biomorphological and Functional Sciences, University of Naples "Federico II", Naples, Italy; 2 Department of Medicine and Surgery, University of Salerno, Italy; 3 Department of Public Health, University of Naples "Federico II", Naples, Italy; 4 Department of Clinical Medicine and Surgery, University of Naples "Federico II", Naples, Italy

## Abstract

**Background:**

Fibrosarcoma (FS) accounts for about 3% of all soft tissue sarcomas. It may arise in any area of the body, but it is relative rare in the head and neck district. Fine-needle cytology (FNC) is widely used in the diagnosis of neoplastic and non-neoplastic lesions of soft tissue. This article describes a case of FS of the neck diagnosed by FNC.

**Methods:**

FNC was performed in a sub-fascial supraclavicular mass of an elderly patient under ultrasound (US) control. FNC was used to prepare cytological smears that were conventionally and immunocytochemically (ICC) stained.

**Results:**

Smears showed a monomorphous spindle cell population and were positive at ICC for Vimentin and negative for CKAE1AE3, Actin, S-100, CD68, CT and PAX-8. The cytological diagnosis was confirmed by histological diagnosis. The patient underwent surgical resection and subsequent radiotherapy.

**Conclusions:**

FNC diagnosis of FS is reliable and accurate and may be conveniently used in the scheduling of surgical procedures, when needed, avoiding the treatment of benign nodules.

## Introduction

Old age is characterized by increased incidence of degenerative and neoplastic diseases. Oxidative stress mechanisms, occurring in response to chemicals, pollutants, and high-caloric diet have been implicated [1-3]. The World Health Organization (2002) defined fibrosarcoma (FS) as a malignant tumor, composed of fibroblasts with variable collagen and, in classical cases, a herringbone architecture [4]. Conventional FS accounts for only 1 to 3% of sarcomas arising from soft tissues [5,6] and falls into two main groups, the adult and infantile types, both very uncommon. Adult FS usually appears in the fourth to sixth decades of life as a painful, deep-seated mass. The extremities (mainly thighs and forearms) and the trunk are the most favored sites with a male predominance [5,6]. These malignancies do not require an extensive vasculature to grow and spread towards distant organs, so that it is unlikely that FS patients display the elevation in the frequency of endothelial progenitor cells that has been reported in highly angiogenic tumors [7-9]. A preoperative pathological diagnosis of soft tissue tumors is very important to assess the therapeutic strategy, especially when FS is located in the head and neck, posing a great diagnostic and therapeutic challenge [10]. The clinical appearance of thyroid cancer is that of a nodules, some time representing a challenging diagnostic dilemma with thyroid or unusual extrathyroidal masses [11,12]. Fine-needle cytology (FNC) has an established role in the diagnosis of various neoplastic and non-neoplastic lesions in different anatomical districts and can be used as an useful alternative to excisional biopsy in the diagnosis of soft tissue tumors [13]. FNC is widely used in the diagnosis of thyroid nodules [12,14-16], lymph nodes [17-21], and salivary glands [22,23]. Moreover, the application of molecular techniques to FNC has dramatically increased its sensitivity and accuracy [16,19,24-34]. Since the head and neck region is characterized by a significant heterogeneity and a variety of organs and pathologies, FNC can provide specific information to define the organ or tissue involved, the specific pathologies and being also useful to the differential diagnosis [32,35,36]. The aim of this study is to present one case of conventional FS in an elderly patient in which FNC pre-surgical diagnosis contributed to a correct and differentiated treatment.

### Materials and methods

A 73-year-old man came to our attention at the outpatient clinic of Cytopathology, Azienda Ospedaliera Universitaria, University of Napoli "Federico II", with a swelling in the right supraclavicular region, which was first noticed two months earlier and doubled in size in a few months. The patient reported a history of long-standing multinodular goiter, but the last ultrasound examination, performed two years before, did not show the mass. At the physical examination, the mass was painful and hard at the touch, with ill-defined edges. Computed tomography (CT) showed a 45 mm sub-fascial mass with indistinct borders, compressing the neurovascular bundle from the right lobe of the thyroid. The US-guided FNC of the mass and on-site evaluation (ROSE) was performed by a cytopathologist [24,30,37]; the diagnostic procedure and its related risks were explained to the patient and an informed consent was obtained. The technical procedure has been previously reported [17,38]. Three passes were performed: smears obtained from the first pass were used to prepare standard cytological smears, which were alcohol-fixed and Papanicolaou stained, and air dried and Diff-Quik stained respectively. Being this latter satisfactory for cellularity at ROSE, two additional passes were used to prepare alcohol-fixed smears for immunocytochemistry (ICC) using a Ventana Benchmark (Ventana Tuxon, AZ). Prediluted monoclonal antibodies used for ICC were the following: Cytokeratin (AE1AE3), Vimentin (VIM), Actin, S-100, CD68, Calcitonin (CT) and PAX-8. Technical procedures have been previously reported [21,33].

## Results

**Figure 1:**
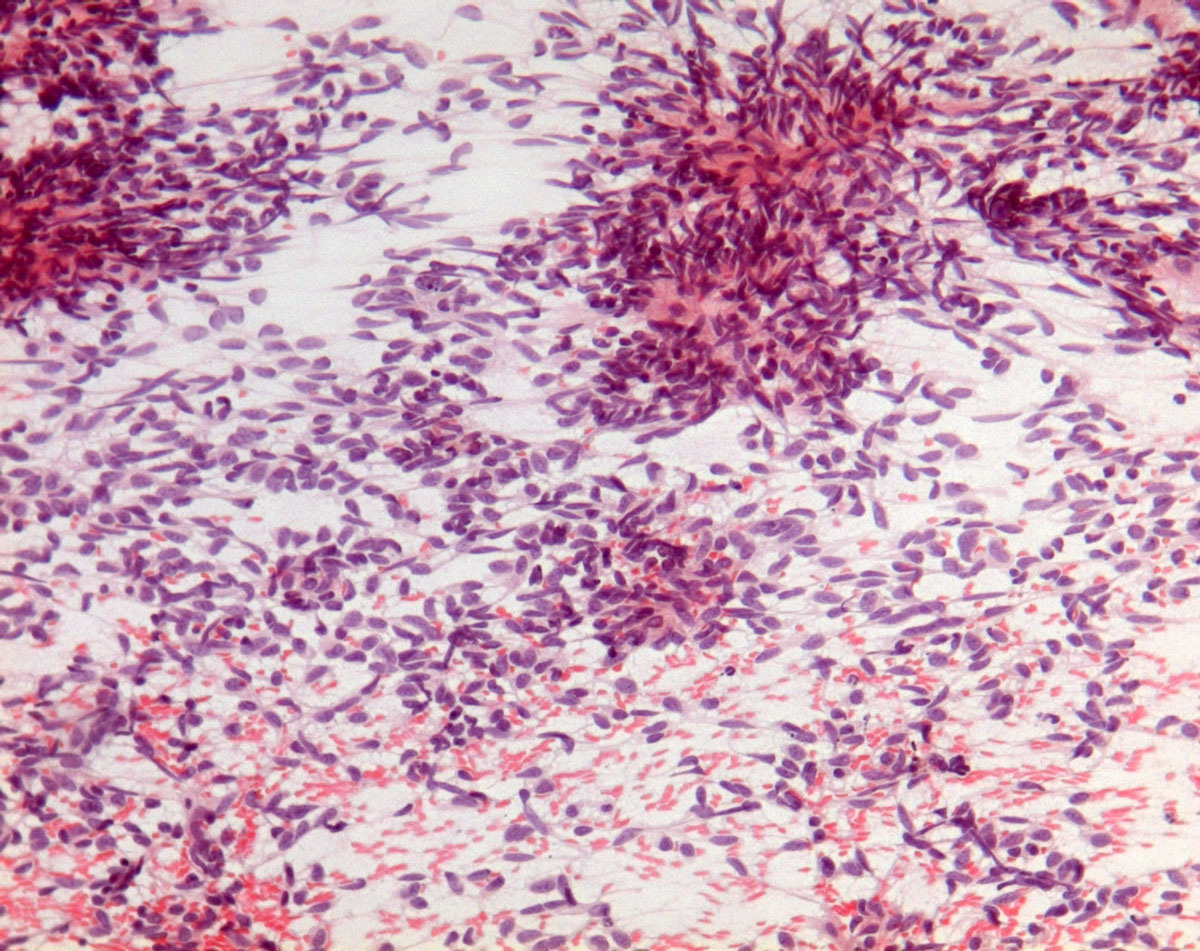
**Aspiration smear shows monomorphic cell population scattered or aggregated in loose cellular groups (Papanicolaou stain 106X)**.

**Figure 2:**
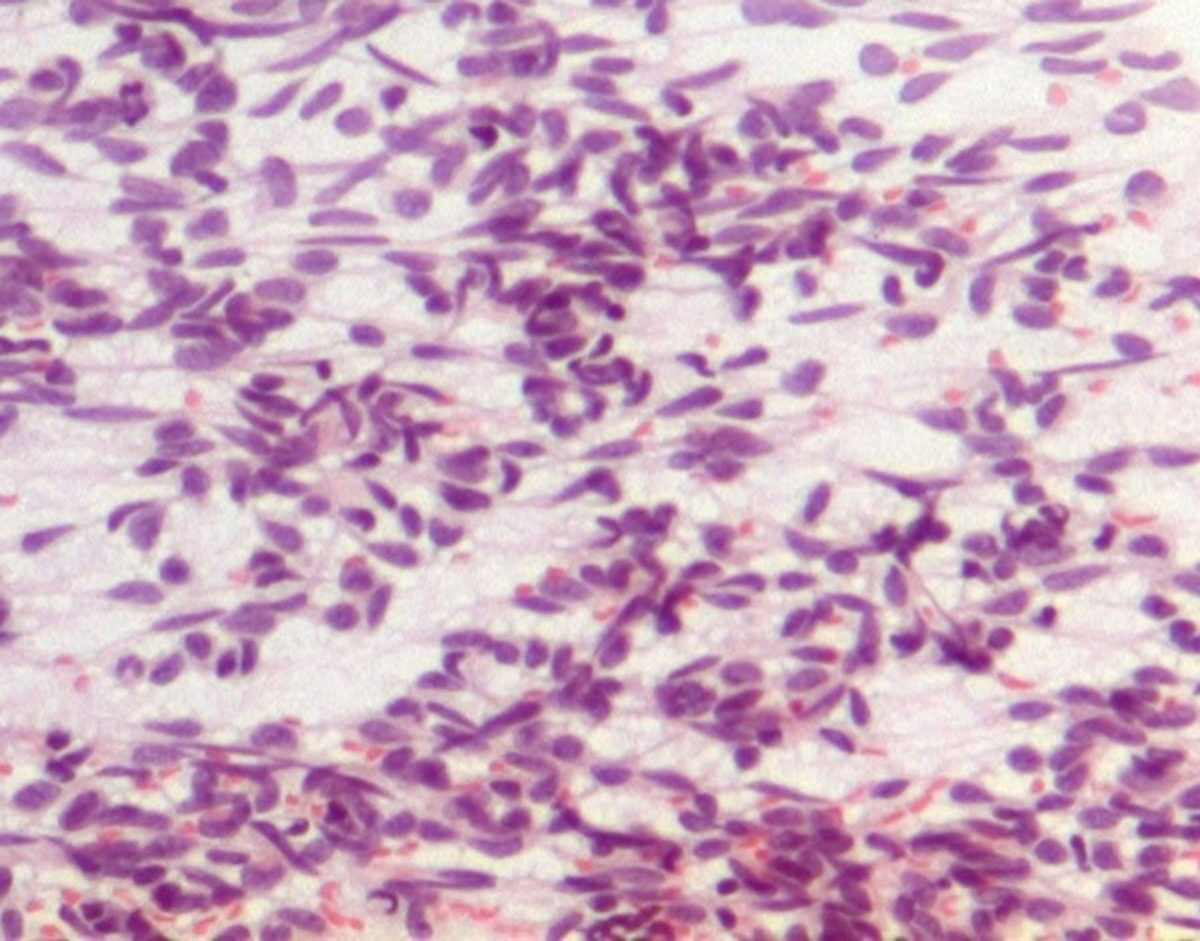
**Spindle-shaped cells with hyperchromatic, fusiform, tapering nuclei and elongated, pale, poorly delimited cytoplasm (Papanicolaou stain 430X)**.

**Figure 3:**
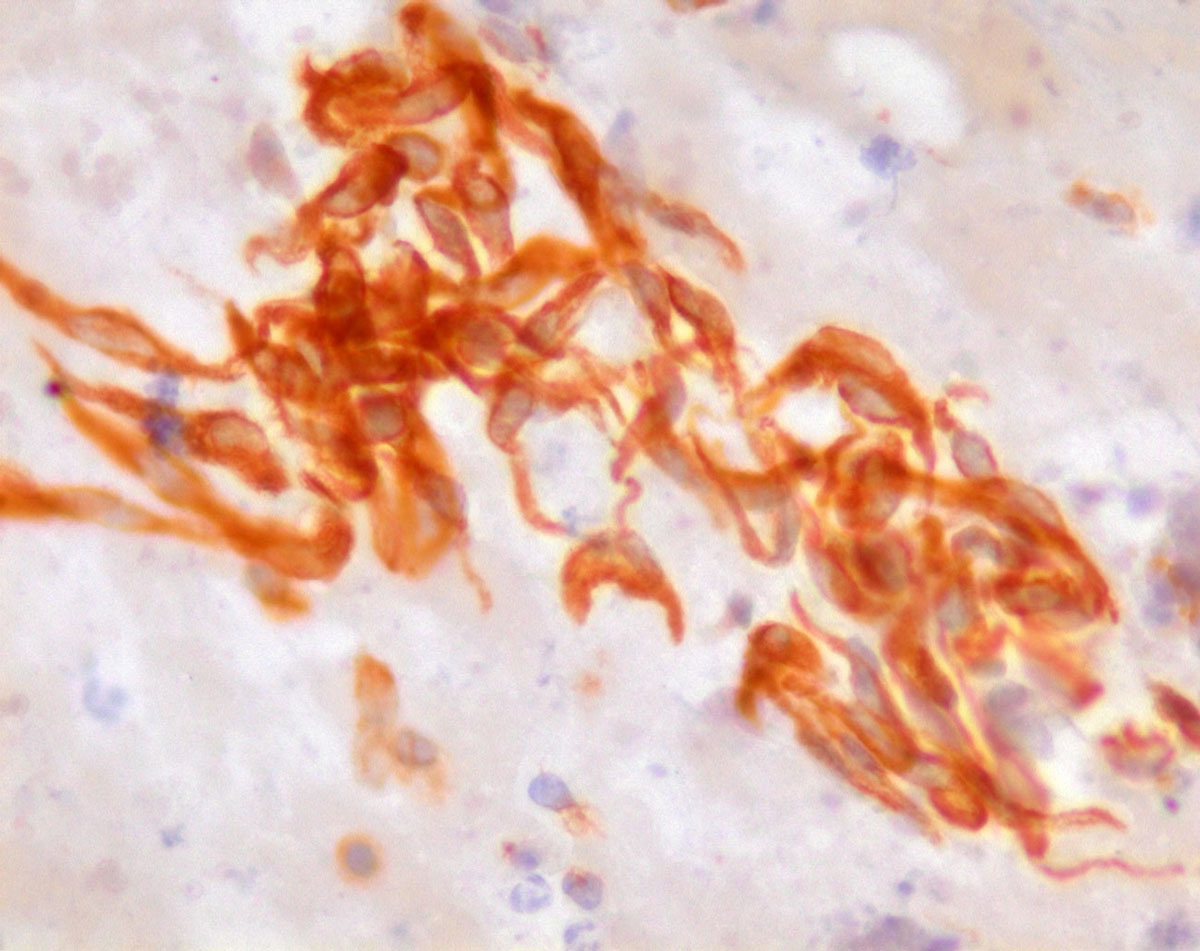
**Spindle cells positive for Vimentin (Immunostain 430X)**.

US evaluation of the neck at the time of the FNC showed an homogeneous hypoechoic mass with ill-defined edges that showed a close relationship with the right thyroid lobe. The smears were very cellular and consisted of a relatively monomorphic cell population scattered or aggregated in loose cellular groups (Figure 1). The cells were spindle-shaped and showed hyperchromatic, fusiform, and tapering nuclei with scanty, elongated and poorly delimited cytoplasm (Figure 2). These cells showed minimal pleomorphism and an inconspicuous mitotic index. ICC showed positivity for VIM (Figure 3) and negativity for CKAE1AE3, Actin, S-100, TG, CT and PAX-8. On the basis of the clinical and instrumental presentation and the cytological features, a differential diagnosis with other spindle cell lesions of the neck was pointed out. Namely, the differential diagnosis included mesenchymal neoplasms and pseudotumors such as nodular fasciitis [15,23,39,40], possible primary thyroid tumors such as the anaplastic carcinoma or medullary carcinoma [15,41], as well as other lesions located in the neck with a spindle cellular component, such as the ectopic thymoma [42,43]. On the basis of morphological and phenotypic features, a diagnosis of differentiated, mesenchymal spindle cell tumors, possibly a FS, was formulated. On the basis of the cytological diagnosis, the mass was excised without removing the thyroid and other anatomical structures. The histological examination confirmed the FNC diagnosis of conventional FS. The patient underwent radiotherapy and, after one year, is alive without signs of disease.

### Discussion

Adult FS is essentially a diagnosis of exclusion from other spindle cell mesenchymal tumors; by definition, it is negative at the immunocytochemistry (IHH) stains for epithelial, myogenous and neural markers, as well as for CD34, CD99, bcl-2 and nuclear beta-catenin [4-6,10]. There is no reported hint of the remodeling of its Ca^2+ ^signaling machinery, which has conversely been described in other types of solid tumors [44-48]. The incidence of conventional FS is greatly diminished with comparison to the past, probably due to the use of IHH that allowed a reclassification of soft tissue tumors with a FS-like morphology, and identified specific variants of FS (Low-grade Fibromyxoid Sarcoma and Sclerosing Epithelioid FS) or FS areas in other pathological entities (Dermatofibrosarcoma protuberans) [5]. With reference to soft tissue lesions, head and neck sarcomas are rare and heterogeneous [10]. FS has also been reported in the head and neck areas and, as in other districts, it has to be differentiated form other benign, locally aggressive and malignant spindle cell tumors. Moreover, in this specific district, FS has to be differentiated from spindle cell tumors of different histogenesis including some thyroidal, salivary glands and lymph-nodes tumors [4,13,15,36,39,40,43,49-51]. Today, the treatment of head and neck soft tissue sarcomas is multidisciplinary, and the surgical approach with an accurate evaluation of the surgical extension remains the main therapeutic procedure [5,10,49,52,53], thus a pre-operative diagnosis should be obtained before any treatment [5,10]. FNC smears, coupled with ancillary techniques, is a recognized technique for the diagnosis of benign and malignant lesions of the soft tissues [36,51,54]. Indeed, the material aspirated through FNC can be used not only for the morphological assessment, but also for ancillary techniques which include ICC, as well as flow cytometry, hybridization techniques, and molecular biology techniques [14,16,20,24,26,32-34,36,42,55]. Nonetheless, the diagnosis of soft tissue tumors on cytological samples is complex and extreme caution is required in the exact classification of spindle-cell tumors by FNC, as this may have a major impact on patient management [5,10,22,36]. Despite the variety of possible mesenchymal and non mesenchymal lesions with a spindle cell cytological pattern, conventional smears allow the evaluation of relative few parameters such as cellularity, cell features, patterns and background; therefore, conventional techniques have to be coupled with ICC, and require a precise clinical history and accurate imaging. In this case, the differential diagnosis includes a broad spectrum of spindle cells lesions and US and CT could not exclude a possible thyroidal origin of the mass. Therefore, among thyroidal neoplasms, the anaplastic thyroid carcinoma [40,51] and the spindle cell variant of medullary carcinoma were considered [41,56]. The first was excluded because of the minimal nuclear atypia, the lack of necrotic and neutrophilic inflammatory components in the smears and the negativity for PAX-8 at the ICC [49]. The negativity for calcitonin at ICC, and not in the serum, ruled out a medullary carcinoma [40]. E1 type ectopic thymoma, an epithelial spindle cell tumor located in the deeper tissues of the neck, was also considered in the differential diagnosis [42,43]. This possibility was also excluded because of the negativity for epithelial antigens (AE1/AE3) at ICC. Finally, other mesenchymal neoplasms that can arise in the neck, such as the Malignant fibrous histiocytoma (MFH) or the schwannoma, were excluded on the basis of the cytological and ICC characteristics [13,36]. Namely, MFH is characterized by an evident nuclear pleomorphism which was not detected in this case. Schwannoma generally shows a fibrilar matrix with spindle cells and nuclear palisading, which were not observed [13,35,36,41]. With reference to the phenotype, the cellular population showed a clear positivity for Vimentin only, being S100 and CD68 negative, which excluded the schwannoma and the MFH, respectively [5,13,35,36,40].

In conclusion, FNC, combined with clinical and imaging data, is a useful procedure for a timely cytological diagnosis of soft tissue tumors, including FS. FNC, together with an adequate ICC, is a feasible and accurate tool, allowing a proper pre-surgery management.

## Competing interests

The authors declare that they have no competing interests.

## Authors' contributions

IC: conception and design, interpretation of data, given final approval of the version to be published; AC, MV, VDS, CC, AG, MPC: acquisition of data, drafting the manuscript, given final approval of the version to be published; IC, MV: critical revision, given final approval of the version to be published.

## Authors' information

IC = Assistant of Pathology at University of Naples "Federico II"

AC = Assistant of Pathology at University of Salerno

VDC = Aggregate Professor of Thoracic Surgery at University of Salerno

MC = Aggregate Professor of Anatomy, University of Naples "Federico II"

CC = Aggregate Professor of Oncology, University of Naples "Federico II"

AG = Aggregate Professor of Pediatric Surgery at University of Salerno

MV = Associate Professor of Endocrinology at University of Salerno

## Declarations

Funding of this supplement came from personal funds.

This article has been published as part of *BMC Surgery *Volume 13 Supplement 2, 2013: Proceedings from the 26th National Congress of the Italian Society of Geriatric Surgery. The full contents of the supplement are available online at http://www.biomedcentral.com/bmcsurg/supplements/13/S2
